# A customized protocol to assess bone quality in the metacarpal head, metacarpal shaft and distal radius: a high resolution peripheral quantitative computed tomography precision study

**DOI:** 10.1186/1471-2474-14-367

**Published:** 2013-12-24

**Authors:** Lynne Feehan, Helen Buie, Linda Li, Heather McKay

**Affiliations:** 1 Department of Physical Therapy, Faculty of Medicine, University of British Columbia (UBC), Vancouver, BC, Canada; 2 The Bone Imaging Laboratory, University of Calgary, Calgary, AB, Canada; 3 Departments of Orthopedics and Family Medicine, Faculty of Medicine, UBC, Vancouver, BC, Canada; 4 Arthritis Research Centre of Canada, Richmond, BC, Canada; 5 Centre for Hip Health and Mobility, Faculty of Medicine, UBC, Vancouver, BC, Canada

**Keywords:** HR-pQCT, Bone microstructure, Volumetric bone mineral density, Precision, Metacarpal head, Metacarpal shaft, Ultra-ultra-distal radius, Early rheumatoid arthritis

## Abstract

**Background:**

High Resolution-Peripheral Quantitative Computed Tomography (HR-pQCT) is an emerging technology for evaluation of bone quality in Rheumatoid Arthritis (RA). However, there are limitations with *standard* HR-pQCT imaging protocols for examination of regions of bone commonly affected in RA. We developed a customized protocol for evaluation of volumetric bone mineral density (vBMD) and microstructure at the metacarpal head (MH), metacarpal shaft (MS) and ultra-ultra-distal (UUD) radius; three sites commonly affected in RA. The purpose was to evaluate short-term measurement precision for bone density and microstructure at these sites.

**Methods:**

12 non-RA participants, individuals likely to have no pre-existing bone damage, consented to participate [8 females, aged 23 to 71 y [median (IQR): 44 (28) y]. The *custom* protocol includes more comfortable/stable positioning and adapted cortical segmentation and direct transformation analysis methods. Dominant arm MH, MS and UUD radius scans were completed on day one; repeated twice (with repositioning) three to seven days later. Short-term precision for repeated measures was explored using intraclass correlational coefficient (ICC), mean coefficient of variation (CV%), root mean square coefficient of variation (RMSCV%) and least significant change (LSC%_95_).

**Results:**

Bone density and microstructure precision was excellent: ICCs varied from 0.88 (MH_2_ trabecular number) to .99 (MS_3_ polar moment of inertia); CV% varied from < 1 (MS_2_ vBMD) to 6 (MS_3_ marrow space diameter); RMSCV% varied from < 1 (MH_2_ full bone vBMD) to 7 (MS_3_ marrow space diameter); and LSC% _95_varied from 2 (MS_2_ full bone vBMD to 21 (MS_3_ marrow space diameter). Cortical porosity measures were the exception; RMSCV% varying from 19 (MS_3_) to 42 (UUD). No scans were stopped for discomfort. 5% (5/104) were repeated due to motion during imaging. 8% (8/104) of final images had motion artifact graded > 3 on 5 point scale.

**Conclusion:**

In our facility, this *custom* protocol extends the potential for in vivo HR-pQCT imaging to assess, with high precision, regional differences in bone quality at three sites commonly affected in RA. Our methods are easy to adopt and we recommend other users of HR-pQCT consider this protocol for further evaluations of its precision and feasibility in their imaging facilities.

## Background

Despite marked improvements in the clinical management of systemic inflammatory joint-disease in early rheumatoid arthritis (RA), people with RA remain at risk for developing underlying systemic inflammatory mediated bone-changes [1-4]. Changes can include progressive periarticular bone thinning (osteopenia) and development of resorptive bone lesions (erosions) [5,6]. Periarticular bone damage, most commonly seen in the bone near the metacarpal phalangeal and wrist joints, can contribute to the development of hand deformities and profound functional limitations in people living with RA [6,7]. Additionally, systemic extra-articular inflammatory bone changes contribute to a two-fold increase in fracture risk with aging in people living with RA [8-11].

Currently, radiography and several clinical imaging systems, such as magnetic resonance imaging (MRI), computed tomography (CT), ultrasonography (US), dual-energy X-ray absorptiometry (DXA) and digital X-ray radiogrammetry (DXR) are used clinically to monitor bone changes in RA [12-17]. While these tools are useful for capturing later macro-structural joint and bone damage that occurs in RA, their abilities to identify the earlier bone microstructural bone changes are poor. Thus, there is an urgent need for new imaging technologies and methods to be developed that can reliably identify and characterize these early changes **before** permanent macro structural bone damage occurs. This is especially important given that early microstructural changes are potentially modifiable if they are reliably identified and treated early.

High Resolution Peripheral Quantitative CT (HR-pQCT; SCANCO Medical AG, Brüttisellen, Switzerland) is a promising imaging technology capable of imaging fine bone internal ‘micro’ detail at a resolution similar to the thickness of a human hair (75 to 100 microns) [18]. Thus, HR-pQCT imaging is a promising tool for evaluating the changes in bone quality that accompany RA. However, research that uses this tool in RA is limited and just emerging [19-32]. Further, it is not possible to compare and synthesize findings from studies in RA that used HR pQCT as image location, acquisition and evaluation procedures are not standardized and vary widely [33].

There are a number of possibilities for these inconsistencies with the primary reason related to applying *standard* protocols developed specifically for one region of interest (ROI) to another ROI without consideration of the technical limitations for doing this. Secondly, although a positioning device is available to support *standard* positioning of the arm, this device is not designed to position and stabilize the hand during imaging near the metacarpal phalangeal or wrist joint regions. Thirdly, *standard* semi-automated image evaluation protocols cannot reliably separate (segment) cortical and trabecular bone compartments in the periarticular metacarpal head and very distal radius bone regions that have very thin cortical shells. This is notable as these regions are commonly affected in inflammatory arthritis [34]. Finally, *standard* image evaluation protocols were not designed to evaluate regions that are comprised primarily of compact lamellar cortical bone such as found in the extra-articular metacarpal mid-shaft region which is also commonly affected in inflammatory arthritis [3,35,36].

Recently, HR pQCT semi-automated image analysis capabilities were advanced to allow more accurate segmentation of the cortical bone compartment [37,38]. This relatively new approach was developed to evaluate regions of bone with a thin cortical shell and therefore overcomes some of the limitations associated with the *standard* imaging protocols. In addition, direct transformation image analyses methods developed for microCT analyses ex vivo were recently adapted to evaluate cortical bone density, morphometry and porosity in vivo*,* using HR-pQCT [38-41]. Importantly, these advances permit evaluation of several micro-structural and macro-structural bone parameters within the integral, trabecular and cortical bone compartments that could not previously be assessed using *standard* HR-pQCT evaluation protocol, in vivo. There is a need, however, to assess the precision of adapted semi-automated cortical compartment segmentation and adapted direct transformation image analyses methods for HR-pQCT assessment in vivo*,* generally and at bone sites commonly affected by RA (e.g. periarticular distal radius and metacarpal head regions and extra-articular metacarpal mid-shaft region).

Therefore, the purpose of this study was to determine the short term precision of an HR-pQCT imaging protocol, in vivo customized for the hand and distal radius. The novel features of this protocol include: 1) comfortable positioning and better stabilization of the head, trunk and upper arm, 2) standardized positioning of the hand and forearm using a custom-made positioning device, and 3) adapted semi-automated cortical segmentation and direct transformation image analyses methods that permit assessment of integral, cortical and trabecular bone macro- and microstructural morphometry and bone mineral density at the Metacarpal Head (MH), Metacarpal Shaft (MS) and the Ultra-Ultra-Distal (UUD) radius bone regions. We use the term Ultra-Ultra-Distal (UUD) radius to differentiate the more distal periarticular distal radius location examined in our study, from the *standard* ultra-distal radius scan location [42]. Our secondary objectives were to explore participant tolerance to the novel positioning protocol as well as rates for re-scanning due to motion during imaging and excessive image motion artifact (e.g. graded > 3 on the manufacturer 5 point rating scale) in the final images [43].

## Methods

This precision study was conducted in a medical imaging research centre setting and received academic institutional ethical approval from the University of British Columbia, Vancouver Canada. Community-dwelling adults were recruited from a large urban metropolitan setting. Participants received no financial remuneration for participation and provided informed consent to participate. With the exception of a physician diagnosis of inflammatory arthritis, participants were not screened for any other self-reported health (e.g. diabetes, osteoporosis) or lifestyle (e.g. smoking, alcohol consumption, physical inactivity) condition that may have affected their bone health. We specifically excluded individuals with a diagnosis of inflammatory arthritis as we were not be able to determine a priori if they may already have underlying macro-structural bone damage in the regions of bone we were examining. Participants were also excluded if they: 1) had any physical condition that would prevent them from sitting motionless with their arm in the scanner supported by a positioning device for up to 6 minutes, 2) had metal or surgical implants in the hand or forearm of interest, 3) were pregnant or possibly pregnant, 4) had sustained a fracture in their dominant arm hand or forearm in the previous 12 months, and 5) were unable to read or understand the consent form.

**Figure 1:**
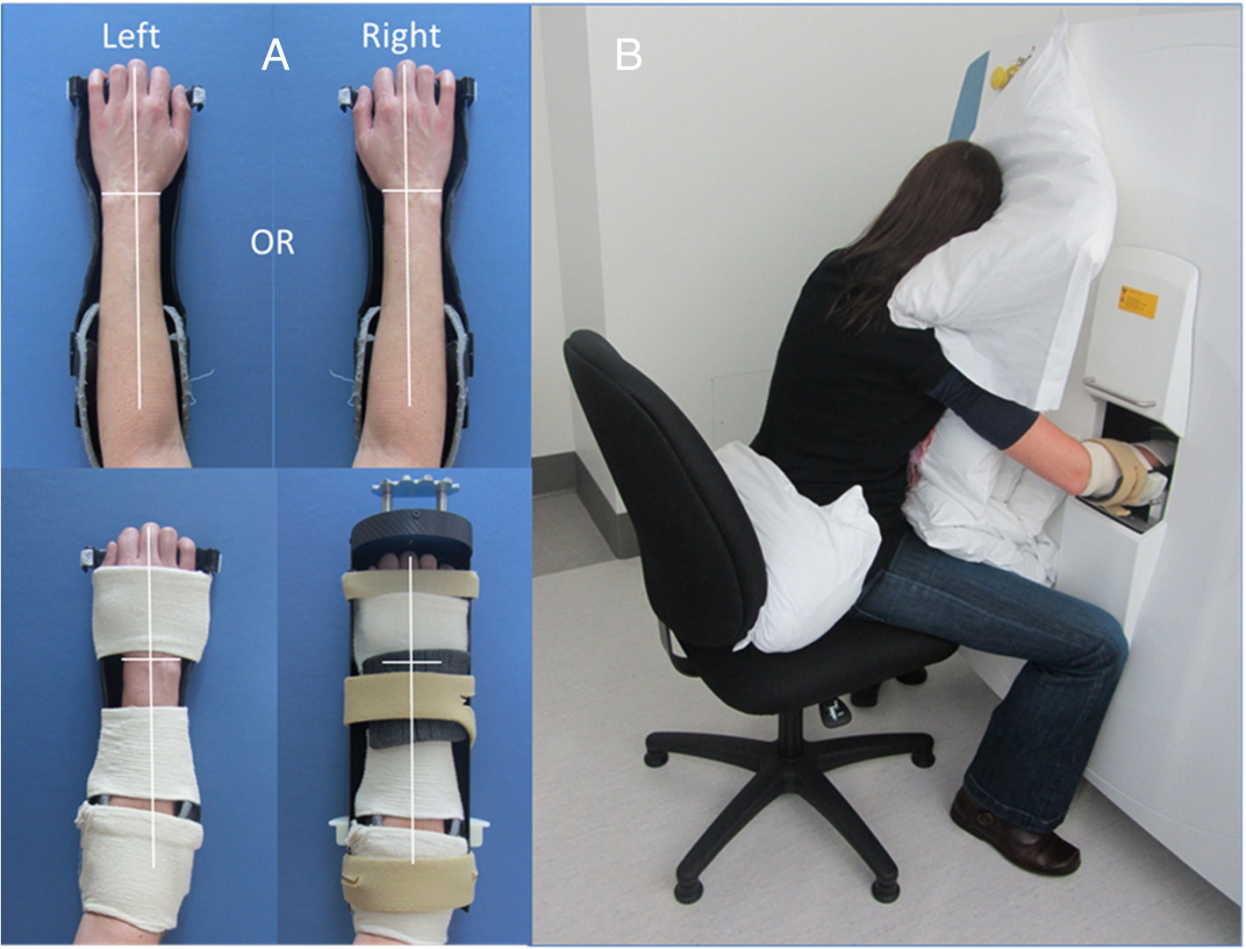
**Custom image acquisition positioning. A)** Shows the standardized positioning of the hand and forearm (left or right) in a custom-made insert (top) with additional stabilization and placement in a modified manufacturer ex-vivo holder (bottom). **B)** Shows the modified positioning for imaging with an individual seated on a chair facing scanner with their head, upper body and opposite arm resting on pillows with the hand to be scanned in the holder and positioned inside the scanner for scanning.

Prior to scanning we assessed height (cm) using a wall mounted stadiometer (SECA corp. Chino, CA) and weight (kg) using a medical grade digital floor scale (Tanita Corporation of America, Inc. Arlington Heights, Ill) using standard techniques. We derived body mass index (BMI) as wt/ht^2^ (kg/m^2^) [44]. Following these anthropometric measures, the hand and forearm were positioned in a custom-made positioning device made of rigid thermoplastic splinting material. The forearm was aligned parallel to the long axis of the splint and the metacarpal phalangeal joints positioned in 0 degrees of flexion. The splint-supported hand and forearm were then positioned within a holder that was modified from manufacturer specifications to suit the hand (Scanco Medical AG, Switzerland). The hand and forearm were then stabilized with additional strapping (Figure 1A). Participants were positioned to face the imaging system. Pillows were placed behind participants’ hips and in front of them so that the participant could lean forward and rest on the pillows with their opposite arm, upper body and head comfortably supported. The holder, with the arm correctly positioned within it, was then placed inside the HR pQCT unit for scan acquisition (Figure 1B).

A single trained operator (author LF) performed all scans using standard in vivo imaging parameters (82 μm nominal isotropic resolution, 60 kV_p_ effective energy, 900 μA current, and 100 ms integration time). The training involved a rigorous and standardized training protocol developed by the facility for the safe operation of the scanner. Manufacturer specifications for the scanner define that for every 110 slices acquired the measurement time is 2.8 minutes with an effective dose of 3 μSv at distal extremity sites. This estimate of effective dose is based on a weighted computed tomography dose index (CTDIw) of 6.1 mGy and a local dose of 3.2 mGy using standard HR-pQCT in vivo image acquisition parameters [45]. A trained operator also performed daily density calibrations and weekly geometry calibrations of the HR-pQCT imaging system using the manufacturer’s calibration phantom.

Three scans of the dominant arm were completed in series during a single scanning session. The ROIs included the metacarpal head (MH), metacarpal mid-shaft (MS) and ultra-ultra-distal (UUD) radius sites. To assess short-term precision with repositioning, we acquired two additional series of three scans with repositioning between each series. The additional two series were completed during a single scanning session, three to seven days after the initial scans.

**Figure 2:**
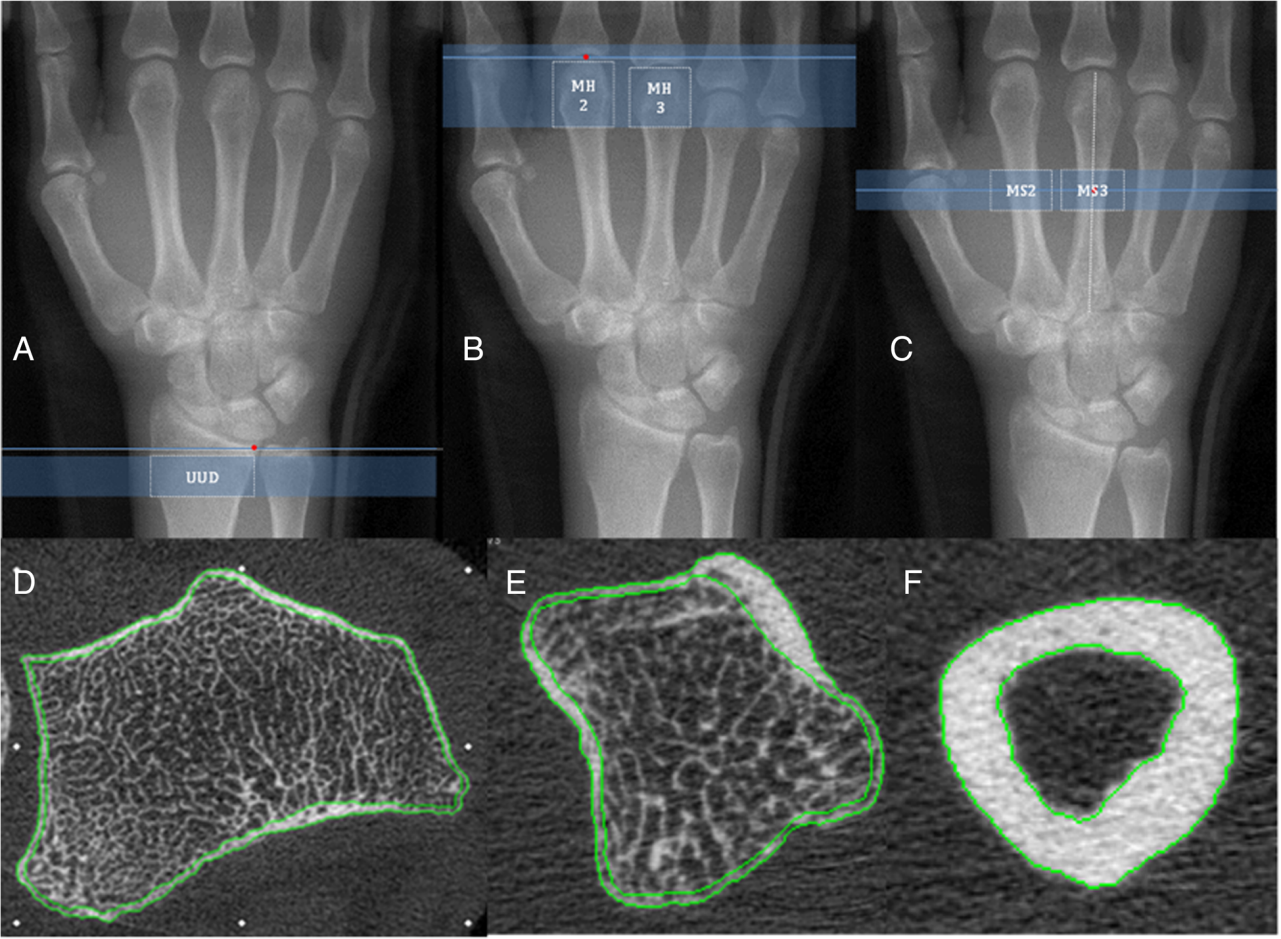
**Scan locations and cortical segmentation.** Top Row **(A,B,C)** shows the reference line, scan location and Region of Interest (ROI) analyses overlaid on a 150 mm scout view for the Ultra-Ultra-Distal Radius **(A)**, Metacarpal Head **(B)** and Metacarpal Shaft **(C)** scans. Bottom Row **(D,E,F)** shows examples of semi-automated cortical compartment segmentation in one HR-pQCT slice for the UUD radius **(D)**, Metacarpal Head **(E)** and Metacarpal shaft **(F)** ROIs.

Prior to each scan, we performed a 150 mm length scout view of the hand and distal forearm which is the maximum available length for a scout view. The reference line for the radius scan was located at the medial edge of the distal radius; the scan region was 1 mm proximal to this reference line and extended 9.02 mm (110 slices) proximally. For the metacarpal head scan, the reference line was the tip of the most distal second or third metacarpal head; the scan started 2 mm distal to this reference line and extended 18.04 mm (220 slices) proximally. For the metacarpal shaft scan, the reference line was half (50%) the total length of the metacarpal shaft assessed on the scout view. The metacarpal shaft scan region of interest extended from 4.5 mm distal to the reference line to 9.02 mm (110 slices) proximal to the reference line (Figure 2 A, B, C).

The operator visually assessed all images for motion artifact at the completion of the three-scan series. If motion artifact was apparent in only one image the operator repeated the scan. If there was motion artifact in two or more of the scans across the series, the operator repeated the scan at one site only. Our image order of priority was the distal radius followed by the metacarpal head.

Images were then independently analysed by 1 of 2 trained and experienced operators, one of whom was the same person as the image acquisition operator in this study (first author LF), the other a study research assistance. Before conducting any image analysis in this study, each operator was required to obtain an intra-rater reliability coefficient (Pearson R) of ≥ 0.90 for measures of UUD trabecular bone fraction from at least 10 images assessed twice by the same operator within 7 to 10 days [46].

Prior to analysis, each image was graded visually for motion artifact using the 5-point manufacture grading system [47]. We included images graded 3 or less by both operators for final data analysis [43]; any disagreement was resolved by consensus. Image analyses were conducted based on operator availability; operators did not use image registration to evaluate repeated scans. Operators were blinded to previous image analyses data; we allowed at least 10 days between image analyses of a repeated scan in any individual by the same operator. Both operators assessed the same numbers of scan images.

Using the manufacturer evaluation software (V 6.0), the operator analyzed five sub-regions of interest [1 - UUD radius (110 slices); 2 - MH2 & MH3 (110 slices); 2 - MS2 and MS3 (110 slices)] (Figure 2, A,B,C). They performed semi-automated contouring of the periosteal bone surface and segmented bone from surrounding soft tissue using standard manufacturer evaluation script protocols [48]. The operator extracted cortical and trabecular regions using the semi-automated segmentation method [37,38], but applied a modified boundary condition for analysis of the metacarpal head.

Following initial segmentation, the operator made minor adjustments to endosteal and periosteal contours as needed [39]. This step included a visual inspection of the computer generated lines for delineation of the cortical region segmentation in all slices, making minor manual corrections to any deviations from accurate periosteal or endosteal surface delineation (Figure 2, D,E,F). Manual correction at this step was rarely indicated; usually only required for the correction of the endosteal edge delineation in a limited number of slices in any image. The most common reason for the need for any manual correction was in instances when there were very larger intra-cortical pores or large bi-cortical breaks created by vascular channels. These manual adjustment procedures have been described in further detail by Burghardt et al., [38].

The operator then ran a series of evaluation scripts using the manufacturer evaluation software for assessment of the full, cortical and trabecular bone regions using direct transformation image analyses scripts adapted from standard microCT evaluation scripts recently developed for cortical bone and described in more detail by Nishiyama KK et al. [40], and Liu XS et al., [41]. These adopted direct transformation evaluation scripts for HR-pQCT are now included in current upgrades of manufacturer evaluation software.

For the periarticular UUD Radius, MH2 and MH3 regions we examined apparent volumetric bone mineral density (vBMD) for the full (vBMD_full_ - mgHA/cm^3^), cortical (vBMD_Cort_ - mgHA/cm^3^) and trabecular (vBMD_Trab_ - mgHA/cm^3^) bone regions. We also examined selected microstructural morphometric bone parameters, including:

•*Cortical bone*: thickness (CtTh - mm) and porosity (CtPo - %).

•*Trabecular bone*: volume fraction (BV/TV_trab_ - %), number (TbN – 1/mm), thickness (TbTh - mm) and separation (TbSp - mm).

At the extra-articular MS2 and MS3 mid-shaft sites we examined full and cortical bone apparent volumetric BMD (vBMD_full_ & vBMD_cort_ - mgHA/cm^3^), as well as, cortical bone material bone mineral density (vTMD_cort_ - mgHA/cm^3^). In addition we examined the following selected micro- and macro-structural morphometric parameters:

•*Full bone*: volume (BV_full -_ mm^3^), volume fraction (BV/TV _full -_ %), section modulus – major direction (SM_full -_ mm^3^), polar moment of inertia (pMOI_full -_ mm^4^), and marrow space diameter (MSdia - mm).

•*Cortical bone*: thickness (CtTh - mm), porosity (CtPo - %), volume (BV_cort -_ mm^3^), volume fraction (BV/TV_cort_ - %), section modulus – major direction (SM_cort -_ mm^3^), polar moment of inertia (pMOI_cort -_ mm^4^).

Direct transformation evaluation methods applied to images acquired using HR-pQCT, in vivo tend to overestimate some trabecular bone outcomes (TbTh, TbSp and BV/TV_trab)_[49,50]. Therefore, the *standard* manufacturer HR-pQCT evaluation script applies a correction factor to these parameters to adjust for known differences. We also applied this correction factor to variables acquired at the UUD Radius, MH2 and MH3 sites so as to directly compare our data with values acquired using *standard* image evaluation methods at other bone regions [41]. Trabecular bone volume fraction (BV/TV_trab_s_) was derived using a standard approach [trabecular bone apparent volumetric bone mineral density (vBMD_trab_) divided by 1200 mg/cm^3^)]. Trabecular thickness (TbTh_s_) and trabecular separation (TbSp_s_) were derived using a standard approach; BV/TV_s_ and 1 – BV/TV_s_ divided by TbN, respectively. Standard evaluation of HR-pQCT images uses direct transformation methods to determine trabecular number (TbN) and full bone and trabecular bone apparent volumetric bone mineral density (vBMD_full_ and vBMD_trab_). Therefore we did not apply conversion factors to these variables.

We assessed short-term precision of repeated measures with repositioning using intraclass correlational coefficient (ICC), mean coefficient of variation (CV%), root mean square coefficient of variation (RMSCV%) and least significant change (LSC%_95_) [51]. Participant tolerance to the imaging protocol and rates of excessive image motion artifact were assessed by percentage of scan reacquisition due to discomfort or motion during imaging and percentage of final images graded as higher than 3 on a 5 point scale respectively [47,52,53].

## Results

12 individuals (8 females) participated. Participants were aged 23 to 71 years [Median (IQR): 44 (28) y]. Participants’ BMI varied from 19 to 30 kg/m^2^ [Median (IQR): 24 (4.5) kg/m^2^] (Table 1). Of the 108 potential scans, 104 were completed (96%). The four scans not completed included 2 MH and MS scans not done in one participant during the second session as the participant was not feeling well and did not want to re-schedule. Of the 104 completed scans, none needed to be stopped due to discomfort during the scanning session. Whereas, 5 of the 104 completed scans (5%; 3 MH, 1 UUD, 1 MS) were repeated at the time of acquisition due to motion artifact detected by the operator at the time of imaging. Of 104 final images acquired, we excluded 8 (8%; 3 UUD, 3 MH, 2 MS) from the final image analyses due to motion artifact graded higher than 3. Notably, of the 8 images excluded from the final analyses, 4 images (1 UUD, 2 MH, 1 MS) were from the same participant (71 y.o. male) who had a resting hand tremor that was not detected at the time of screening [43,53,54]*.* This left 96 images available for final analyses.

**Table 1:** Participant demographics

	**Females (n = 8)**	**Males (n = 4)**	**All (n = 12)**
Age (years): median (IQR); min-max	44 (n/a); 23-62	45 (n/a); 23-71	44 (28); 23-71
Height (cm): median (IQR); min-max	165 (n/a); 158-174	185 (n/a); 175-195	173 (18.5); 158-195
Weight (kg): median (IQR); min-max	64 (n/a); 63-76	77 (n/a); 64-94	65 (12.5); 55-94
BMI (kg/m^2^): median (IQR); min-max	24 (n/a); 19-30	21 (n/a); 19-24	24 (4.5); 19-30

For the final repeated measures analyses we were able to analyze imaging data at the UUD region for 11 of the 12 participants as data from one participant was excluded due to motion artifact in 2 of the 3 UUD images. For the metacarpal head and shaft regions, we analyzed data from 10 of the 12 participants. One participant’s MH and MS repeated measures data was missing because these scans were not completed during the follow up session. As well, one other participant’s MH and another participant’s MS data were excluded due to motion artifact in 2 of 3 images.

Precision for measures of volumetric BMD and macro- and microstructural bone morphometry was very high at all five sub-ROIs [51,55]. ICCs varied from 0.88 (MH2 - TbN) to .99 (MS3 - pMOI_cort_). CV% varied from < 1 (MS2 - vBMD_cort_) to 6 (MS3 – Ms_dia_). RMSCV% varied from < 1 (MH2- vBMD_full_) to 7 (MS3- MS_dia_) and LSC%_95_ varied from 2 (MS2 - vTMD_cort_) to 21 (MS2 - MS_dia_). The exceptions were the poor measures we report for cortical porosity at all three measurement sites [RMSCV% varying from 19 (MS3) to 42 (UUD)] (Tables 2,3,4).

**Table 2:** Summary of the results for Ultra-Ultra-Distal (UUD) radius region of interest (n = 11)

**Variables**				**Mean (SD)**	**ICC (0.000-1.000)**	**Mean coefficient of variation (CV%)**	**Root mean square CV (RMSCV%)**	**Least significant change% (LSC%**_**95**_**)**
Density (Apparent)	Full Bone (mgHA/cm3)	vBMD_full_	D & S	362 (102)	0.986	2.3	3.2	8.8
	Cortical density (mgHA/cm3)	vBMD_cort_	D	944 (189)	0.962	3.5	3.7	10.4
	Trabecular density (mgHA/cm3)	vBMD_trab_	D & S	272 (69)	0.993	1.3	1.6	4.5
Cortical bone	Thickness (mm)	CtTh	D	0.59 (0.20)	0.959	5.9	7.3	20.2
	Porosity (%)	CtPo	D	1.3 (0.7)	0.320	34.0	41.7	115.6
Trabecular bone	Bone volume Fraction (%)	BV/TV_trab_	D	37 (7)	0.990	1.7	2.0	5.7
		BV/TV_trabs_	S	23 (6)				
	Number (1/mm)	TbN	D & S	2.4 (0.3)	0.908	3.1	4.3	12.1
	Thickness (mm)	TbTh	D	0.22 (0.02)	0.956	0.79	4.5	12.6
		TbTh_s_	S	0.10 (0.01)				
	Separation (mm)	TbSp	D	0.38 (0.07)	0.932	3.4	1.1	3.0
		TbSp_s_	S	0.34 (0.07)				

D = Direct Transformation Method; S (Grey fill) = Derived Standard Clinical Equivalent.

**Table 3:** Summary of results for the Metacarpal Head (MH) 2 & 3 regions of interest (n = 10)

**Variables**				**Mean (SD)**		**ICC (0.000-1.000)**		**Mean coefficient of variation (CV%)**		**Root mean square CV (RMSCV%)**		**Least significant change% (LSC%95)**	
				**MH3**	**MH2**	**MH3**	**MH2**	**MH3**	**MH2**	**MH3**	**MH2**	**MH3**	**MH2**
Density (Apparent)	Full bone (mgHA/cm3)	vBMD_full_	D & S	438 (89)	434 (82)	0.997	0.998	0.83	0.57	1.0	0.78	2.8	2.2
	Cortical (mgHA/cm3)	vBMD_cort_	D	743 (106)	751 (101)	0.975	0.975	1.5	1.3	2.1	1.6	6.0	4.5
	Trabecular (mgHA/cm3)	vBMD_trab_	D & S	387 (79)	378 (73)	0.996	0.997	0.8	0.7	1.2	1.0	3.2	2.9
Cortical bone	Thickness (mm)	CtTh	D	0.39 (0.07)	0.39 (0.06)	0.933	0.973	4.8	1.6	6.17	2.7	17.1	7.4
	Porosity (%)	CtPo	D	1.2 (0.7)	1.2 (0.4)	0.153	0.727	23.2	17.0	33.2	22.2	92.1	61.4
Trabecular bone	Volume fraction (%)	BV/TV_trab_	D	46 (5)	46 (6)	0.984	0.984	1.2	1.3	1.5	1.6	4.3	4.5
		BV/TV_trabs_	S	26 (13)	26 (13)								
	Number (1/mm)	TbN	D & S	2.6 (0.24)	2.5 (0.23)	0.904	0.884	2.1	2.2	2.5	2.8	6.9	7.7
	Thickness (mm)	TbTh	D	0.23 (0.02)	0.24 (0.02)	0.978	0.978	0.74	1.1	3.9	3.5	10.8	9.8
		TbTh_s_	S	0.11 (0.03)	0.11 (0.03)								
	Separation (mm)	TbSp	D	0.34 (0.05)	0.34 (0.06)	0.928	0.944	3.0	3.0	1.1	1.3	3.0	3.5
		TbSp_s_	S	0.27 (0.05)	0.29 (0.05)								

D = Direct Transformation Method; S (Grey fill) = Derived Standard Clinical Equivalent.

**Table 4:** Summary of results for Metacarpal Shaft (MS) 2 & 3 regions of interest (n = 10)

**Variables**			**Mean (SD)**		**ICC (0.000-1.000)**		**Mean coefficient of variation (CV%)**		**Root mean square CV (RMSCV%)**		**Least significant change% (LSC%**_**95**_**)**	
			**MS3**	**MS2**	**MS3**	**MS2**	**MS3**	**MS2**	**MS3**	**MS2**	**MS3**	**MS2**
Density (Apparent)	Full bone (mgHA/cm3)	vBMD_full_	1181 (207)	1230 (180)	0.994	0.981	0.88	1.8	1.2	2.9	3.3	7.9
	Cortical (mgHA/cm3)	vBMD_cort_	1482 (173)	1492 (172)	0.993	0.996	0.83	0.49	1.0	0.80	2.8	2.2
Density (Material)	Cortical (mgHA/cm3)	vTMD_cort_	1568 (194)	1564 (199)	0.996	0.997	0.57	0.66	0.72	1.0	2.0	2.8
Cortical bone	Thickness (mm)	CtTh	1.8 (0.37)	2.0 (0.34)	0.989	0.989	1.5	1.7	1.9	1.9	5.3	5.1
	Porosity (%)	CtPo	0.29 (0.31)	0.31 (0.22)	0.790	0.949	16.7	29.0	18.8	36.0	52.2	99.7
	Volume (mm^3^)	BV_cort_	374 (101)	412 (109)	0.998	0.998	0.97	0.93	1.3	1.1	3.6	3.1
	Section modulus-major (mm^3^)	SM_cort_	55 (20)	61 (23)	0.998	0.997	1.2	1.4	1.4	1.7	3.9	4.9
	Polar moment of inertia (mm^4^)	pMOI_cort_	476 (234)	535 (256)	0.999	0.998	0.87	1.9	1	2.0	2.8	5.6
Full bone	Volume (mm^3^)	BV_full_	475 (120)	499 (126)	0.997	0.997	0.77	0.97	1.0	1.1	2.8	3.1
	Volume fraction (%)	BV/TV_full_	76 (9)	80 (7)	0.991	0.976	0.71	1.2	0.82	1.5	2.3	4.2
	Marrow space diameter (mm)	MSdia	2.6 (0.87)	2.6 (0.73)	0.961	0.979	5.7	3.9	7.4	4.9	20.5	13.6
	Section modulus – major (mm^3^)	SM_full_	52 (20)	58 (23)	0.997	0.998	1.4	1.1	1.7	1.5	4.6	4.0
	Polar moment of inertia (mm^4^)	pMOI_full_	444 (232)	498 (254)	0.998	0.998	1.3	1.7	1.6	1.8	4.3	5.1

Across all regions, vBMD measurement precision was better than precision for measures of microstructural morphology; RMSCV% for _V_BMD varied from < 1 to 4 compared with microstructural morphology which varied from < 1 to 7. At the periarticular UUD radius and the second and third MH sites, precision was better for trabecular bone microstructural morphology (RMSCV%: < 1 to 4) compared to measures of cortical thickness (RMSCV%: 3 to 7). At the extra-articular second and third MS sites the precision for measures of full and cortical bone density as well as macro- and microstructural morphometry (RMSCV%: < 1 to 3) was better than precision for measures of marrow space diameter (RMSCV%: 5 to 7) (Tables 2,3,4).

## Discussion

This study extends the literature that uses in HR pQCT to examine ‘‘bone quality” in vivo in a novel way using customized image acquisition and analyses protocols to assess bone parameters in the distal forearm and hand. We deliberately focus upon these regions of interest given they are sites where trabecular and cortical bone is commonly affected in individuals living with RA. We demonstrated that our *custom* HR-pQCT imaging protocol, in vivo is a precise means to assess integral, cortical and trabecular bone density and macro- and microstructure (with the exception of cortical porosity) at the MH, MS and UUD radius in our imaging facility.

Some distinguishing features of our *custom* image acquisition methods are; 1) more comfortable and stable positioning of the head, trunk and arm during imaging, and 2) standardized positioning and stabilization of the metacarpal phalangeal and wrist joints in a custom-made positioning device. These are important advantages as better stabilization during imaging reduces the potential for participant motion during scanning as well as the degree of motion artifact in final images. Notably, the percentage of scans repeated due to motion identified at the time of scanning (scan re-acquisition: 5% vs. 29%) as well as percentage of images graded higher than 3 (Poor Image Quality: 8% vs. 20%) was markedly lower than previously reported values for these parameters using the *standard* HR-pQCT distal radius protocol [47,54]. Moreover, standardized positioning allows more consistent visual land-marking to locate the scan ROI. This negates the need for the operator to use computer assisted image registration methods to evaluate repeated images of the same bone regions in either short term follow up or longer term prospective studies [54].

**Figure 3:**
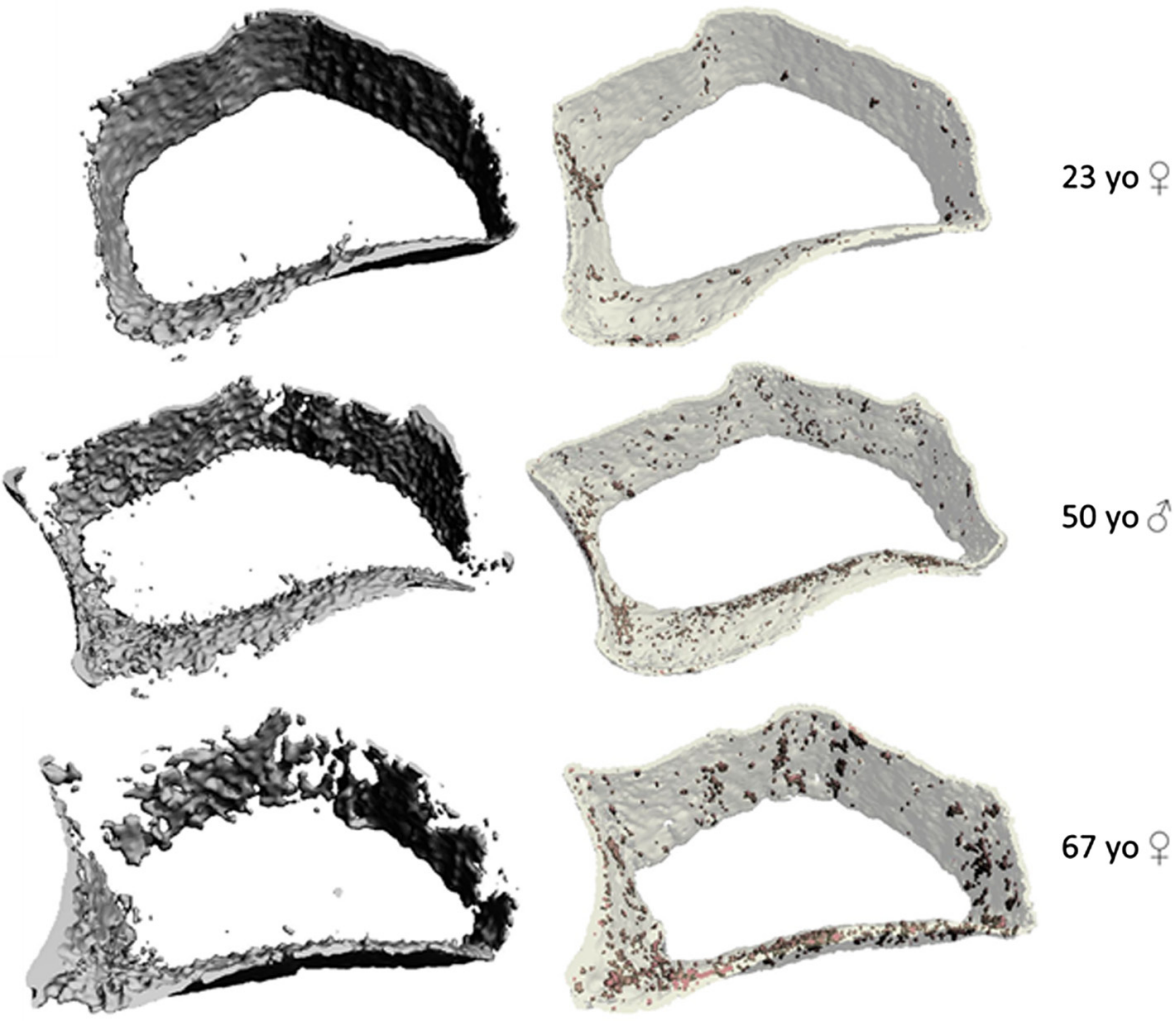
**Cortical compartment 3-dimentional reconstructed images.** 3-D reconstructed images of segmented cortical compartments from the same HR-pQCT images of the ultra-ultra-distal radius region in three participants (top row - 23 y.o. female, middle row - 50 y.o. male, bottom row - 67 y.o. female) using the standard clinical evaluation protocol (left) compared to our semi-automated cortical segmentation protocol (right). The images on the right also show shaded areas of cortical porosity identified with the adapted direct transformation cortical evaluation script.

Using adapted semi-automated cortical segmentation methods ensured the operator was able to reliably extract the cortical bone compartment in all the regions of bone we examined. This is an important finding, especially given the challenges presented by very thin and highly porous cortical shells in the periarticular distal radius and metacarpal head regions (Figure 3). Reliable and more accurate cortical bone segmentation methods add to the unique ability of HR-pQCT imaging, in vivo to evaluate the independent contribution of trabecular and cortical bone compartment density and microstructural parameters to integral bone strength [56-58].

We demonstrated that adapted direct transformation image analysis methods traditionally used in microCT imaging were also able to precisely assess many aspects of integral, trabecular and cortical bone density, macro- and microstructure that are not currently assessed using *standard* HR-pQCT evaluation methods, in vivo (cortical porosity was the exception). By including derived *standard* evaluation equivalent values for trabecular bone volume fraction, thickness and spacing, users are also able to compare outcomes with normative or other values reported at *standard* distal radius and tibia scan sites [59].

Importantly, precision for bone density, macro- and microstructure at the MH, MS and UUD radius regions we report in our imaging facility was comparable to previously reported values for distal radius and metacarpal head bone microstructure and bone mineral density measurement precision using HR pQCT [21,53]. To our knowledge, our study is the first to assess HR-pQCT’s ability to precisely evaluate bone density, bone macro- and microstructure at the very distal periarticular UUD radius site, in vivo. Our findings align with estimates of HR-pQCT precision error, in vivo at the *standard* radius site [RMSCV%; vBMD, < 1–2; microstructure, 1–6] and a site more proximal to the *standard* distal radius location [RMSCV% <1 – 2; microstructure <1-7] [42,60]. As well as HR-pQCT microstructure precision (CV%, < 1–6) at the UUD radius site in cadaver bone ex vivo (similar to the site we assessed) [61]. This is notable as motion artifact is not an issue when assessing tissue, ex vivo.

The metacarpal mid-shaft region provides a unique opportunity to use HR-pQCT imaging to examine cortical bone density and morphometry in vivo in the shaft region of long bone that macro-structurally has a relatively thick (approximately 2 mm) cortical compartment that is comprised primarily of lamellar compact cortical bone. To our knowledge, no other study has examined the precision of HR pQCT for in vivo measures in the mid-shaft region of a long bone. It is encouraging that, with the exception of marrow space diameter and cortical porosity, that apparent and material volumetric bone mineral density, as well as, several macro- and microstructure parameters in this novel mid-shaft region can be assessed with very high precision (RMSCV% < 2) using HR-pQCT, in vivo. Development of novel approaches for evaluation of cortical bone quality is key given the important contribution of cortical bone to overall bone strength and fracture risk, as well as, differences in the rate and mechanisms for cortical and trabecular bone turnover with aging and many chronic diseases [57,62-65].

A few others have examined measurement precision of metacarpal head microstructure in those with RA [21,26,30,31]. Fouque-Aubert et al. [21], used *standard* image methods to assess HR-pQCT density and reported microstructure measurement precision in vivo at the metacarpal head in people living with RA compared with Non-RA controls. They found no notable difference in vBMD measurement precision between those with RA and controls (CV% < 2). These values align exactly with the CV we report for vBMD at the metacarpal head. Fouque-Aubert et al. [21], also found no differences between those with RA and controls for measurement precision of *standard* trabecular microstructural parameters (CV varied from 3 to 7%), with the exception of trabecular separation (CV of 13% in RA participants versus 6% in controls). Comparably, the CV for *standard* and other additional microstructural parameters we examined at the metacarpal head varied from 1 to 5%. As our protocol aimed to control motion artifact due to robust stabilization of the measured part, standardize positioning of the hand and wrist joints and enhance accuracy of segmentation of the very thin cortical bone compartment – these factors taken together may account for improved cortical and trabecular bone microstructure measurement precision at the metacarpal head in this study compared to the *standard* imaging protocols reported previously [21].

Cortical porosity is difficult to assess reliably and this held true for all regions of bone examined in our study. Relatively low precision for cortical porosity measured at the *standard* radius (RMSCV; 13%) [38], and a more proximal distal radius site (RMSCV; 6 +/− 8%) have been reported previously [42]. Precision for cortical porosity at the MH, MS and UUD radius sites we examined were even poorer (RMSCV, 19 - 42%). There are a number of factors that might explain this. First, is the current 82 μm image voxel resolution of HR-pQCT, in vivo*.* Thus, it is difficult to resolve pore diameters smaller than this within the intra-cortical bone region, particularly in regions of bone with very thin cortical shells [66,67]. Second, on the endosteal surface of the cortical-trabecular bone interface, cortical pores are difficult to distinguish from marrow space [39]. One clear solution is enhanced image resolution in vivo*.* Indeed, as better image resolution continues to evolve and newer methods of cortical porosity evaluation are developed more precise methods to assess porosity in regions of thin or more compact cortical bone locations will become available [68,69].

We acknowledge that our study has limitations. This study was conducted in a small cohort of health adults in a single imaging facility, using imaging operators with extensive experience with in vivo image acquisition and analyses using HR-pQCT. As such, the precision of this custom protocol in our facility cannot be generalized to other imaging facilities that utilize HR-pQCT imaging that are not familiar with, or trained in, the image acquisition and analyses protocols used in this study. Further studies, ideally from multiple centres, are required to further define the precision and feasibility for this protocol. We also could not explore inter-rater reliability as none of the images in this study were evaluated by both image analyses operators. However, and notably, the effect of any measurement error associated with individual variations in image analyses was likely negligible given the high measurement precision demonstrated in this study. As well, we excluded people living with inflammatory arthritis in this precision study as we wanted to explore the utility of our *custom* HR-pQCT protocol for identifying and characterizing early microstructural bone changes in bone prior to permanent macro-structural damage occurring. This was an a priori decision as we were unable to determine if a person diagnosed with inflammatory arthritis may or may not already have underlying bone changes in the regions of bone commonly affected by RA. As such, our findings for measurement precision in the metacarpal head and UUD radius periarticular regions cannot be generalized to individuals living with more advanced RA where macro-structural changes from resorptive bone lesions (erosions) may already be present or where positioning may be affected by the presence of hand deformities. We also did not apply newly available cortical bone porosity image analyses procedures so we do not know if they would enhance the precision of cortical porosity measures at the MH, MS or UUD radius [68,69].

In summary, we demonstrated excellent precision for measures of bone density and many macro- and micro-structural parameters at the MH, MS and UUD radius using a customized HR-pQCT protocol in our facility. The novel image acquisition protocol was well tolerated by all the participants and provided excellent stabilization of the forearm and hand during imaging resulting in a low percentage of final images with excessive motion artifact. The novel image acquisition protocol reflects a number of other practical advantages over the *standard* distal radius image acquisition protocol and can be easily adopted by HR-pQCT users. Additionally, the adapted semi-automatic cortical segmentation and direct transformation image evaluation methods used in this study are also available to other HR-pQCT users through the most recent manufacturer image evaluation software upgrades.

## Conclusion

In our facility, this *custom* protocol extends the potential for using in vivo HR pQCT imaging technology to assess, with high precision, integral, trabecular and cortical bone density and microstructure at sites in the distal forearm and hand most commonly affected in rheumatoid arthritis. As such, we recommend that this customized protocol be considered by other HR-pQCT users for further evaluations of its precision and feasibility in their imaging facility.

## Competing interests

The authors declare that they have no competing interests.

## Authors’ contributions

LF conceived the study and design, managed and participated in the image acquisition and analyses, contributed to the data analysis and interpretation and drafted all versions of the manuscript. HB was involved in the development and implementation of the adapted image analyses protocols. LL and HM contributed to the study design, interpretation of the data and editing of manuscript drafts. All authors read and approved the final manuscript.

## Pre-publication history

The pre-publication history for this paper can be accessed here:

http://www.biomedcentral.com/1471-2474/14/367/prepub
